# Skeleton and Glucose Metabolism: A Bone-Pancreas Loop

**DOI:** 10.1155/2015/758148

**Published:** 2015-03-19

**Authors:** Maria Felicia Faienza, Vincenza Luce, Annamaria Ventura, Graziana Colaianni, Silvia Colucci, Luciano Cavallo, Maria Grano, Giacomina Brunetti

**Affiliations:** ^1^Section of Pediatrics, Department of Biomedical Sciences and Human Oncology, University of Bari “A. Moro”, 70124 Bari, Italy; ^2^Section of Human Anatomy and Histology, Department of Basic Medical Sciences, Neurosciences and Sense Organs, University of Bari, 70124 Bari, Italy

## Abstract

Bone has been considered a structure essential for mobility, calcium homeostasis, and hematopoietic function. Recent advances in bone biology have highlighted the importance of skeleton as an endocrine organ which regulates some metabolic pathways, in particular, insulin signaling and glucose tolerance. This review will point out the role of bone as an endocrine “gland” and, specifically, of bone-specific proteins, as the osteocalcin (Ocn), and proteins involved in bone remodeling, as osteoprotegerin, in the regulation of insulin function and glucose metabolism.

## 1. Introduction

Bone is a dynamic structure that is constantly subject to remodeling by specialized cells, the osteoclasts (OCs), osteoblasts (OBs), and osteocytes. Bone remodeling consists of removal of mineralized bone tissue by OCs, to leave a resorptive cavity filled by the migration of OB precursors which differentiate into mature OBs. Osteocytes regulate both remodeling and mineralization processes and represent the terminal stage of the OB lineage embedded in the bone matrix. Osteocytes are also the source of molecules which control the production and activity of OCs, such as osteoprotegerin (OPG) and Receptor activator of nuclear factor kappa-B ligand (RANKL) [1].

Recently, bone has emerged as an endocrine “gland,” and some key mediators of this alternative function have been identified.

This review focuses on the role of the skeleton as endocrine organ, its modulation of glucose tolerance by secretion of bone-specific proteins, in particular the osteocalcin (Ocn), and how proteins involved in bone remodeling, like OPG, are associated with impairment of insulin function.

## 2. The Role of Insulin in Regulating the Functions of Bone Cells

The regulation of glucose metabolism occurs through the interplay of multiple hormones which operate in many target organs. Insulin plays an important role in glucose regulation by promoting glucose uptake in adipose tissue and muscle and by suppressing gluconeogenesis in liver. To perform these functions, insulin binds to its receptor (InsR), a tyrosine kinase expressed in hepatocytes, adipocytes, myoblasts, and OBs.

However, deletion of the InsR in muscle, the most important site of glucose uptake, does not affect blood glucose levels, insulin concentration, and glucose tolerance, suggesting that other tissues, like bone, could be involved in glucose regulation [2, 3].

Insulin has been demonstrated to be an osteogenic hormone both* in vitro* and* in vivo*. OBs express abundant insulin receptors and respond to insulin treatment [4–6] by increasing cell proliferation [7, 8], collagen synthesis [5, 9–11], and glucose uptake [12, 13]. Mice knocked out for InsR in their OBs have decreased trabecular bone volume due to reduced bone formation and poor numbers of OBs [3, 14]. In addition, these mutant mice show the reduction of OC erosion depth and low serum levels of cross-linked C-telopeptide (CTX) which indicate a decline of OC activity. Moreover, the treatment with insulin has been shown to be effective in determining the reversibility of skeletal alterations of rodent model with type 1 diabetes and also favoring the healing of fractures [15–19]. Based on these data, there are emerging studies which regard the skeleton as an important regulator of energy metabolism.

## 3. Osteocalcin and Glucose Metabolism: The Bone-Pancreas Loop

Recent investigations, particularly from the Karsenty group, have identified a crucial role for the Ocn in regulating insulin metabolism in a hormonal way [14]. Ocn is the major noncollagen protein secreted by the OBs and it is stored in the extracellular matrix of bone. Before its secretion, Ocn is carboxylated at the level of three Gla residues. This process of carboxylation confers high-affinity binding to hydroxyapatite, the mineral present in bone, and the attachment of carboxylated Ocn to the bone matrix [20]. Instead, when Ocn is uncarboxylated, its binding to hydroxyapatite is reduced, promoting the passage of Ocn into circulation. The involvement of undercarboxylated form of Ocn in a bone-pancreas loop has been demonstrated by previous studies. Ocn-deficient mice show few *β* cells, great fat mass, and decreased insulin sensitivity [21]. Conversely, the subcutaneous infusion of recombinant Ocn into wild-type mice enhances glucose tolerance and improves insulin sensitivity [22].

**Figure 1:**
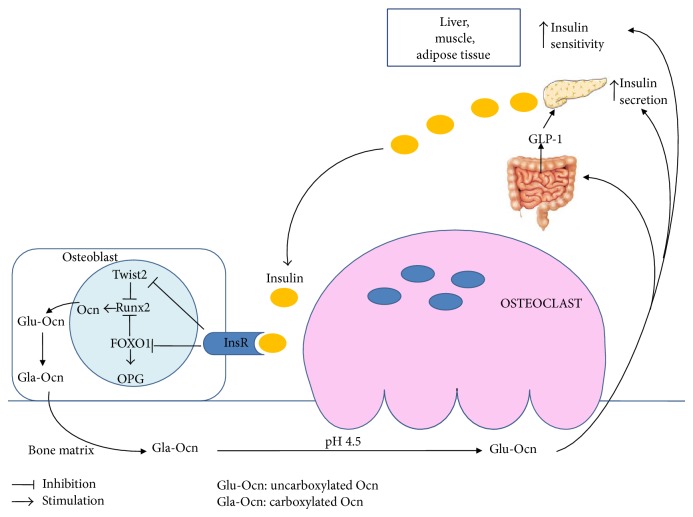
Interplay between Ocn and insulin secretion/sensitivity.

The decarboxylation of Ocn is dependent on bone resorption: insulin signaling in OBs favors the differentiation of OCs and the formation of resorption lacunae by inhibiting the expression of OPG [14]. The low pH present within these lacunae promotes the decarboxylation of Ocn and consequently its activation [14] (Figure 1). Conversely, a tyrosine phosphatase produced by* Esp* (*Ptprv*) gene blocks Ocn decarboxylation and decreases serum levels of active form of Ocn [21]. The human ortholog of* Esp* (OST-PTP, also called osteotesticular protein tyrosine phosphatase) is not active in humans but recent studies have shown that there are additional tyrosine phosphatases, such as TC-PTP1, expressed in OBs [21–24]. These phosphatases can regulate Ocn activity and glucose homeostasis by acting on the insulin signaling pathway in the OBs [21, 23, 24].

### 3.1. Uncarboxylated Osteocalcin Functions

The regulation of systemic glucose metabolism and insulin resistance by Ocn occurs in a hormonal manner [25].

Firstly, Ocn stimulates insulin secretion by *β*-cells both directly [26, 27] and indirectly promoting the secretion of gut glucagon-like peptide-1 (GLP-1) [28] (Figure 1). The effects of Ocn on activating ERK and insulin secretion are mediated by Ocn receptor, an orphan receptor belonging to the C family of GPCRs, highly expressed in the mouse pancreatic *β*-cell line [29]. The Ocn-GPRC6A network has strong physiological effects in the mouse, but the clinical relevance of this endocrine pathway in humans is less certain. Up till now, no mutations or polymorphisms of* Osteocalcin* or* GPRC6A* genes have been reported in humans [27]. Secondly, Ocn promotes *β*-cell proliferation by increasing Ccnd2 and Cdk4 expression in *β*-cells [22]. Thirdly, Ocn increases insulin sensitivity in liver, muscle, and adipose tissue (Figure 1) by upregulation of adiponectin gene expression in adipocytes [21].

InsR signaling in OBs has a double and positive action on Ocn. On one side, InsR induces* Osteocalcin* gene expression in OBs by blocking the negative activity of the nuclear factor Twist2 on Runx2, the master gene of OB differentiation and Ocn expression [30]. Furthermore, InsR signal decreases the ability of FOXO1 to activate the OPG promoter (Figure 1), thus reducing the secretion of this inhibitor of OC function by OBs [31].

### 3.2. Clinical Relevance of Osteocalcin Glucose Regulation

A number of studies have established that numerous aspects of Ocn biology are similar in rodents and humans. There are several data indicating that serum levels of uncarboxylated Ocn negatively correlate with insulin resistance, obesity, diabetes, or markers of the metabolic syndrome (MetS) [32–35]. Interestingly, important weight loss causes a decrease of insulin resistance as well as an increase in Ocn levels in obese children [36], and acute aerobic exercise could increase serum uncarboxylated Ocn in obese subjects [37]. Furthermore, serum Ocn has also been positively correlated with improved glucose control in subjects with type 2 diabetes [38]. Women with gestational diabetes show high Ocn levels which correlate with insulin secretion parameters and return to normal values postpartum [39]. This raising of serum Ocn levels could represent an adaptive process to counteract glucose intolerance during gestational diabetes.

## 4. Osteoprotegerin

OPG is a soluble glycoprotein belonging to the tumor necrosis factor receptor superfamily which decreases bone resorption by inhibiting the differentiation and activation of OCs [40]. It acts as a decoy soluble receptor for RANKL, thus preventing RANKL binding with its receptor RANK on OCs, thus inhibiting osteoclastogenesis [41]. RANKL/RANK/OPG system mediates important and complex relations between the vascular, skeletal, and immune systems [42, 43]. OPG is mainly secreted by bone but it is produced also by different tissues, including endothelial and smooth muscle cells [43]. OPG improves endothelial cells survival but it may induce endothelial inflammation and proliferation of endothelial and vascular smooth muscle cells, thus promoting atherogenesis. OPG knockout mice show osteoporosis and vascular calcification, reintroducing the hypothesis that metabolic bone diseases and vascular diseases, for example, arterial calcification, share common pathways [44, 45]. OPG administration prevents calcification induced by Warfarin or high doses of vitamin D in rats, but the effects of OPG in humans are different from those in rodents [46]. In humans, high OPG levels have been found in patients with type 2 diabetes, coronary artery diseases, hypothyroidism, hypercholesterolemia, and obesity, as well as in aging men [47–51]. A population-based study has demonstrated that high serum OPG represents an independent risk factor for the progression of atherosclerosis, as well as of vascular mortality [52]. On the other hand, results of experimental studies suggest that OPG has also vasoprotective properties through reduction of vascular calcification [53]. Recent data have indicated a role of OPG as metabolic biomarker [54]. In obese subjects, OPG has been found to be positively associated with insulin resistance [55, 56]. Furthermore, high OPG levels have been associated with risk of metabolic syndrome and microvascular complications in type 2 diabetes patients [57].

## 5. Other Regulators of Bone-Pancreas Loop

### 5.1. Vitamin D

Vitamin D is recognized as a key regulator of bone and mineral metabolism. Vitamin D signaling is mediated by binding of the physiologically active form 1*α*,25- dihydroxyvitamin D3 (1,25D3) to its intracellular receptor (VDR) which, after translocation to the nucleus, binds to vitamin D response elements (VDREs) of target genes involved in different pathways (cell proliferation, differentiation, and immunomodulation) [58].

1,25D3 has an indirect effect on bone formation through intestinal and renal regulation of calcium levels. However, the presence of VDRs in OBs suggests a direct role of vitamin D in bone metabolism, supported by gene expression profiling studies examining mRNA in OBs treated with 1,25D3 [59–62]. Moreover, data from* in vitro* and* in vivo* models have shown that 1,25D3 can exert catabolic or anabolic actions on bone, depending on species and/or environmental context, in order to control the plasma calcium homeostasis [63]. In particular, 1,25D3 showed stimulatory effects on human and rat OBs and inhibitory effects on murine OBs. Generally, in condition of negative calcium balance, VDR signaling in OBs enhances bone resorption stimulating the expression of RANKL [64] and suppresses bone mineralization by inducing expression of Ocn and osteopontin [65, 66].

The identification of VDRs in different organs and tissues including the prostate, brain, colon, breast, immune cells, and pancreas underlines the extra skeletal effects of vitamin D [67]. In particular, vitamin D regulates glucose homeostasis and insulin secretion by binding to its VDR in pancreatic *β*-cells [68]. Vitamin D deficiency has been associated with insulin resistance in nondiabetic subjects and with a reduced insulin production in type 2 diabetics [69].

The role of vitamin D in regulation of insulin production by pancreatic *β*-cells is supported by the presence of VDREs in the human* InsR* gene promoter [70]. Moreover, several studies have shown that polymorphisms of* VDR* gene may affect insulin release and insulin sensitivity [71, 72]. In addition, pancreatic *β*-cells express a plasma membrane VDR, which seems to mediate an insulinotropic rapid effect of vitamin D, independent of mRNA transcription and protein translation [73].

### 5.2. Gastric Inhibitory Polypeptide (GIP)

Gastric inhibitory polypeptide (GIP) is a 42-amino-acid hormone, secreted from K cells of duodenum and proximal jejunum. The main function of GIP is the stimulation of the postprandial insulin secretion from the pancreatic islets [74]. GIP exerts its effects by binding to the GIP receptor (GIPR) and stimulates insulin secretion by *β*-cells in a glucose-dependent manner [75]. GIPRs are present on OBs, OCs, osteocytes, and chondrocytes [76, 77] and GIP signaling has an anabolic action on bone. In fact, several studies using* in vitro* and animal models demonstrated an antiapoptotic and stimulating effect on OBs [76, 78, 79] and a direct antiresorptive activity probably mediated by cAMP. [77]. GIP is designed as a member of the “entero-osseous axis,” responsible for the postprandial reduction of bone resorption [78, 80]. This is supported by a recent study of Nissen et al. showing a reduction of CTX plasma levels after infusion with GIP, both during euglycemia and hyperglycemia [81].

### 5.3. Adiponectin

Adiponectin is a 28 kDa protein produced by differentiated adipocytes and is abundantly present in plasma [82–84]. The biological actions of adiponectin are mediated through the two adiponectin receptors (AdipoR) 1 and 2 and comprise regulation of glucose and lipid metabolism, inflammation, and energy balance [85].

Adiponectin controls glucose homeostasis by enhancing insulin sensitivity and maintaining a functional *β*-cell mass [86]. In particular, adiponectin stimulates muscle glucose utilization [87, 88] and exerts a cytoprotective and antiapoptotic effect on *β*-cells [89]. Moreover, adiponectin influences bone metabolism, even if the mechanisms mediating this effect are controversial.* In vitro* experiments showed that adiponectin promotes proliferation of OBs in human [90] and inhibits osteoclastogenesis, increasing bone mass [91].

Conversely, Shinoda et al. [92] demonstrated that high level of circulating adiponectin represents a risk factor for fractures independent of body composition and BMD [92].

This effect could be the consequence of the stimulation of RANKL and inhibition of OPG expression by adiponectin in OBs [93].

Moreover, a recent study has shown that adiponectin inhibits OB proliferation and induces OB apoptosis in young animals, whereas in older animals it increases the bone mass [94]. Thus, according to this study, adiponectin has opposite influences on bone mass, a local negative action on OBs (inhibition of OB proliferation and induction of OB apoptosis), and an indirect effect through a central signaling that decreases sympathetic tone, leading to increase of bone formation and bone mass [94].

## 6. Conclusions

Recent advances highlighted the role of the bone in modulating metabolic functions. The identification of Ocn as a hormone that stimulates insulin sensitivity in peripheral tissues and insulin secretion by the pancreas has opened the way for new fields of research. Nevertheless, the interactions between bone, pancreas, and probably other organs need to be further explored. There are conflicting results on the effects of antiresorptive drugs for osteoporosis, like bisphosphonates and denosumab, on glucose metabolism. Bisphosphonates and denosumab reduce circulating levels of total Ocn and in particular of the undercarboxylated, active form. However, although in mouse models the suppression of bone turnover with antiresorptive drugs determines important effects on fasting glucose, weight, and diabetes incidence, randomized placebo-controlled trials have demonstrated that the reduction of bone turnover and low levels of undercarboxylated Ocn are not involved in the regulation of insulin sensitivity in humans. Thus, patients receiving such osteoporosis treatments would not be at risk of impaired glucose metabolism or diabetes.

These observations suggest that the bone pancreas loop is more complex than currently known and additional studies will be necessary to evaluate the impact of the connection between the skeleton and metabolism in humans.

Development of new drugs that simultaneously target the skeleton, the glucose metabolism, and the adipose tissue are certain to be considered a future perspective.

Insulin signaling in OBs decreases the expression of OPG, inhibiting FOXO1, and induces Ocn expression, blocking the negative activity of Twist2 on Runx2. Reduction of OPG favors the differentiation of OCs and the low pH of resorption lacunae promotes the decarboxylation of Ocn and consequently its activation. The undercarboxylated Ocn was released into the circulation and stimulates *β*-cells insulin secretion both directly and indirectly by promoting the secretion of gut GLP-1. Moreover, active Ocn increases insulin sensitivity in liver, muscle, and adipose tissue.
